# Warburg Micro syndrome is caused by RAB18 deficiency or dysregulation

**DOI:** 10.1098/rsob.150047

**Published:** 2015-06-10

**Authors:** Mark T. Handley, Sarah M. Carpanini, Girish R. Mali, Duska J. Sidjanin, Irene A. Aligianis, Ian J. Jackson, David R. FitzPatrick

**Affiliations:** 1 MRC Human Genetics Unit, Institute of Genetics and Molecular Medicine, University of Edinburgh, Edinburgh EH4 2XU, UK; 2 Division of Neurobiology, The Roslin Institute and R(D)SVS, University of Edinburgh, Easter Bush, Midlothian EH25 9RG, UK; 3 Department of Cell Biology, Neurobiology, and Anatomy, Medical College of Wisconsin, Milwaukee, WI 53226, USA

**Keywords:** RAB18, Rab, Ras, GAP, GEF

## Abstract

*RAB18*, *RAB3GAP1*, *RAB3GAP2* and *TBC1D20* are each mutated in Warburg Micro syndrome, a rare autosomal recessive multisystem disorder. RAB3GAP1 and RAB3GAP2 form a binary ‘RAB3GAP’ complex that functions as a guanine-nucleotide exchange factor (GEF) for RAB18, whereas TBC1D20 shows modest RAB18 GTPase-activating (GAP) activity *in vitro*. Here, we show that in the absence of functional RAB3GAP or TBC1D20, the level, localization and dynamics of cellular RAB18 is altered. In cell lines where TBC1D20 is absent from the endoplasmic reticulum (ER), RAB18 becomes more stably ER-associated and less cytosolic than in control cells. These data suggest that RAB18 is a physiological substrate of TBC1D20 and contribute to a model in which a Rab-GAP can be essential for the activity of a target Rab. Together with previous reports, this indicates that Warburg Micro syndrome can be caused directly by loss of RAB18, or indirectly through loss of RAB18 regulators RAB3GAP or TBC1D20.

## 1. Background

Warburg Micro syndrome (MIM 600118, 614225, 615222, 615663) is a rare autosomal recessive disorder characterized by severe eye and brain abnormalities [1,2]. It can be caused by loss-of-function mutations in *RAB18*, *RAB3GAP1*, *RAB3GAP2* or *TBC1D20* [3–6]. Importantly, a mutation in any one of the known disease genes produces a clinically indistinguishable condition [6,7]. Affected children are born with cataracts and other eye abnormalities including microphthalmia, microcornea and atonic pupils. They develop postnatal-onset microcephaly and have cerebral malformations that include hypogenesis of the corpus callosum and polymicrogyria. There is usually significant developmental delay and affected individuals are normally unable to sit independently, walk or talk.

Rab proteins are small GTPases of the Ras superfamily (reviewed in [8]). Proteins in this class are often referred to as molecular switches because they adopt different conformations depending on whether they are bound to GDP or GTP. It is thought that in a GDP-bound state they are largely inactive, whereas in a GTP-bound state they are able to mediate downstream effects by interacting with binding proteins referred to as effectors. The switching between these states is governed by two classes of regulatory protein: the guanine-nucleotide exchange factors (GEFs), which mediate the exchange of bound GDP for GTP, and the GTPase-activating proteins (GAPs), which stimulate the GTP hydrolysis activity of their substrate GTPase(s). Rab proteins also undergo cycles of membrane association and dissociation that accompany their cycles of GTP binding and hydrolysis. This is accomplished via GDP-dissociation inhibitor (GDI) proteins that mediate the extraction of membrane-associated, GDP-bound Rabs into the cytosol. The proper retargeting of cytosolic Rab proteins back onto cellular membranes requires GDI and may also require a GDI-displacement factor (GDF).

RAB3GAP1 and RAB3GAP2 were characterized as forming a complex with GAP activity towards Rab3 isoforms before their involvement in Micro syndrome, or that of RAB18, was known [9,10]. Recent work has now shown that the complex also functions as a RAB18GEF [11]. Given that the symptoms of individuals lacking a functional RAB3GAP1 or RAB3GAP2 mimic those of individuals lacking functional RAB18, this implies that in the absence of cellular RAB18GEF activity, RAB18 is unable to fulfil its cellular role.

The relationship between TBC1D20 and the other disease gene products has not been explicitly explored. However, multiple observations link its function to that of the other proteins and suggest that, like RAB3GAP, it might regulate RAB18. First, mice with a loss-of-function mutation in *Tbc1d20* show a highly similar ocular phenotype to *Rab18*^(−/−)^ mice, exhibiting nuclear cataracts [6,12,13]. Further, at the cellular level, patient fibroblasts, TBC1D20 or RAB18-deficient mouse fibroblasts, and Cos7 cells in which the disease genes have been knocked down all show similarly altered lipid droplet (LD) formation with respect to controls [6,11,12]. Finally, although characterized as a GAP for Rab1 and Rab2 isoforms, TBC1D20 shows modest *in vitro* GAP activity towards RAB18 [14].

In this report, we show that RAB18 can be targeted from the cytosol to the *cis*-Golgi in human fibroblasts, where it can then be stabilized by RAB3GAP/RAB18GEF. Further, we show that TBC1D20 functions to promote RAB18 dissociation from the endoplasmic reticulum (ER) membrane into the cytosol, probably though stimulating RAB18 GTP-hydrolysis.

## 2. Results and discussion

### 2.1. Loss of the RAB3GAP complex or TBC1D20 leads to an increase in cellular levels of RAB18

**Figure 1.**
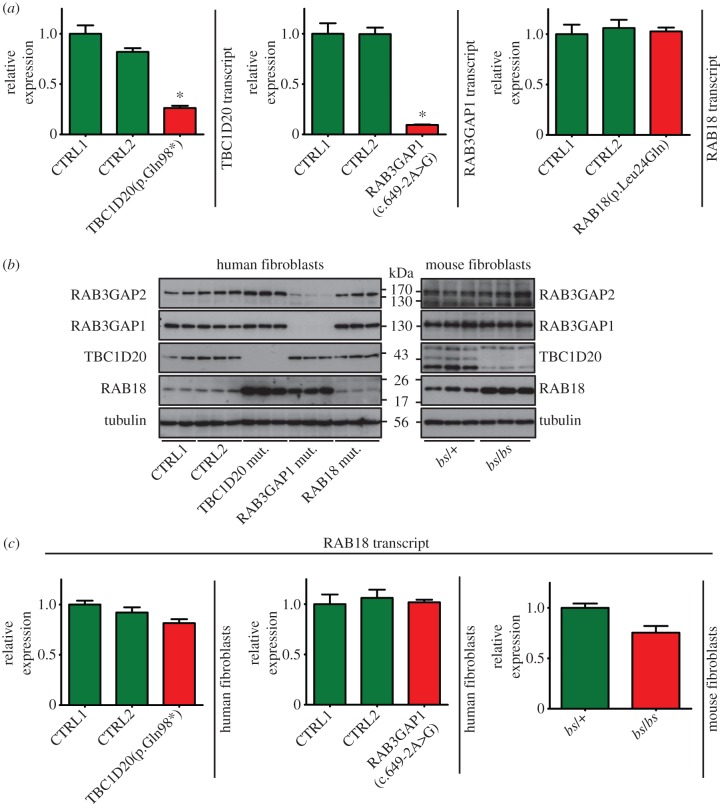
Loss-of-function mutations in *TBC1D20* or *RAB3GAP1* are associated with increased levels of RAB18 protein but not transcript. (*a*) Quantitative RT-PCR (qPCR) shows that levels of TBC1D20 and RAB3GAP transcripts are reduced compared to controls in TBC1D20(p.Gln98*) and RAB3GAP1(c.649-2A>G) patient fibroblasts, respectively, but that levels of RAB18 transcript in RAB18(p.Leu24Gln) fibroblasts are comparable. (*b*) Western blotting shows levels of RAB3GAP1, RAB3GAP2, TBC1D20 and RAB18 protein in control and patient fibroblasts and *blind-sterile* fibroblasts. Blotting for tubulin serves as a control. Each lane on the blots shown corresponds to an individual lysate sample, and each blot is representative of at least three independent experiments. (*c*) qPCR shows that levels of RAB18 transcript are comparable in control and mutant cell lines. qPCR data shown are derived from analysis of at least three cDNAs per genotype, each analysed in triplicate. Primers were designed using the Universal ProbeLibrary Assay Design Center (Roche) and are listed in the electronic supplementary material, table S1. Error bars represent s.e.m. *p < 0.005, unpaired Student's *t*-test.

We began quantitative PCR (qPCR) and western blot analysis of a panel of control and patient-derived human skin fibroblasts [6,11] to determine the effect of mutations in *RAB3GAP1*, *TBC1D20* and *RAB18* on transcript and protein levels of all of the known Micro syndrome disease genes (figure 1). As expected, reduced levels of the cognate transcripts resulted from the splicing mutation in *RAB3GAP1*, RAB3GAP1(c.649-2A>G), and the nonsense mutation in *TBC1D20*, TBC1D20(p.Gln98*), when compared with controls (figure 1*a*). This is consistent with these mutations introducing premature stop codons and thereby rendering the transcripts susceptible to nonsense-mediated decay (NMD). Consistent with this, no RAB3GAP1 or TBC1D20 protein was detectable by western blot in respective cell lines, suggesting that the mutations completely abolish protein expression (figure 1*b*). The missense mutation in *RAB18*, RAB18(p.Leu24Gln), ablates nucleotide-binding [4] but did not affect levels of RAB18 transcript.

Two apparent *trans*-acting post-transcriptional effects were observed. First, we saw that RAB3GAP2 protein was almost undetectable in RAB3GAP1(c.649-2A>G) cells (figure 1*b*). This was not unexpected as a corresponding effect has been reported in *Rab3gap1*^(−/−)^ mice and in Cos7 and HeLa cells in which RAB3GAP1 was knocked down [11,15]. A reasonable explanation for this is that RAB3GAP1 and RAB3GAP2 function as a complex and that RAB3GAP1 is required to maintain RAB3GAP2 stability [15]. Next, we observed that levels of RAB18 were elevated in both RAB3GAP1(c.649-2A>G) and TBC1D20(p.Gln98*) cells (figure 1*b* and the electronic supplementary material, figure S1*a*). The levels of RAB18 transcript were comparable between each cell line and controls (figure 1*c*).

To further explore the effects of loss of TBC1D20 on levels of RAB18, we compared immortalized mouse embryonic fibroblasts (mEFs) heterozygous and homozygous for the ‘*blind-sterile*’ (*bs*) *Tbc1d20* loss-of-function mutation TBC1D20 p.[Phe231Met; p.Arg232 _Val235del] [6]. As in the patient fibroblasts, an increase in levels of RAB18 was observed (figure 1*b* and the electronic supplementary material, figure S1*a*). The levels of *Rab18* transcript were comparable to those in controls (figure 1*c*). Furthermore, when either the human or mouse TBC1D20-deficient cells were treated with cycloheximide to inhibit protein synthesis, the reduction in RAB18 levels over time was slowed compared with controls. This suggests that RAB18 is less rapidly degraded in these cells (electronic supplementary material, figure S1*b,c*). In treated RAB3GAP1(c.649-2A>G) cells, reduction in RAB18 levels occurred at a similar rate to that in controls (electronic supplementary material, figure 1*b*). This suggests that any alteration in RAB18 degradation in these cells is more subtle than in the TBC1D20-deficient lines. Levels of the TBC1D20 substrate RAB1A were unchanged in TBC1D20(p.Gln98*) cells and *bs* mEFs when compared with controls (electronic supplementary material, figure S1*d*).

### 2.2. The RAB3GAP complex stabilizes RAB18 at the *cis*-Golgi in human fibroblasts

**Figure 2.**
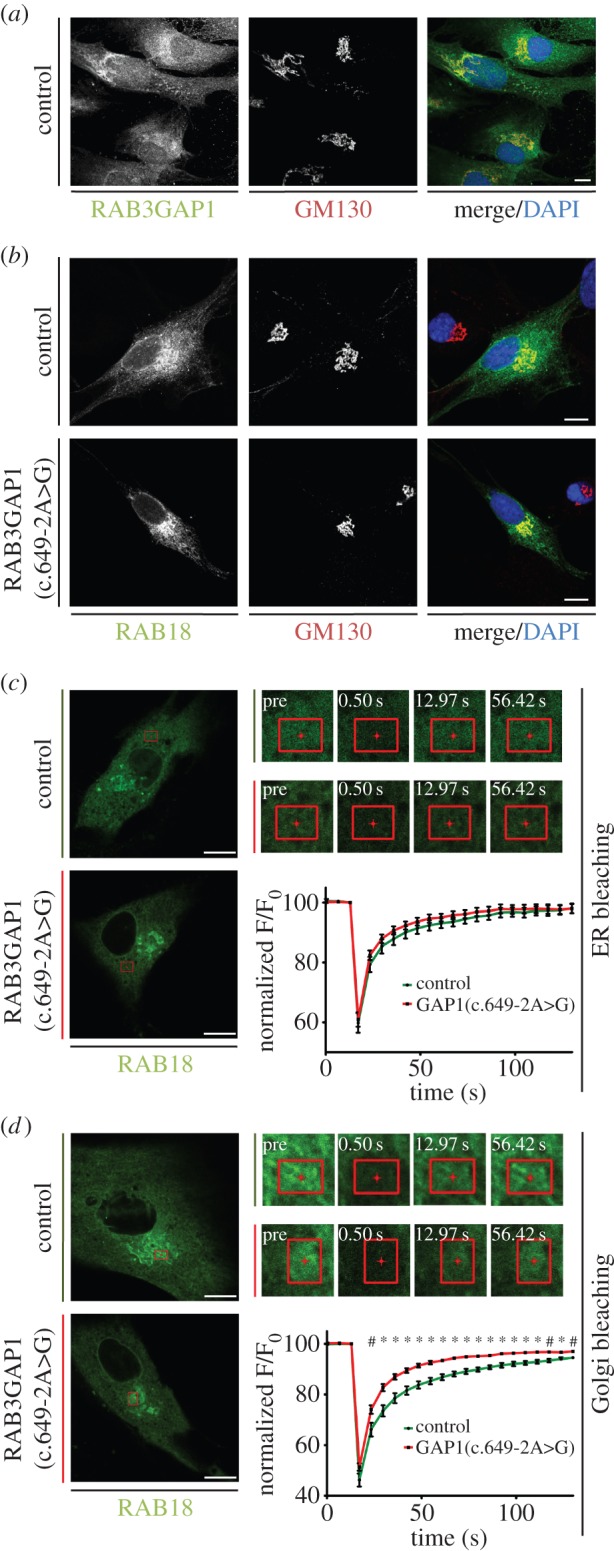
RAB18 dynamics at the Golgi are altered in RAB3GAP1(c.649-2A>G) fibroblasts. (*a*) RAB3GAP1 colocalizes with staining for GM130 in control human fibroblasts. (*b*) GFP-RAB18 colocalizes with staining for GM130 in control and RAB3GAP1(c.649-2A>G) fibroblasts. (*c*) GFP-RAB18 dynamics at the ER in control and RAB3GAP1(c.649-2A>G) fibroblasts. (*d*) GFP-RAB18 dynamics at the Golgi in control and RAB3GAP1(c.649-2A>G) fibroblasts. Indicated ROIs in each cell were bleached with high-intensity laser. Fluorescence recovery in the ROI was recorded over time and normalized with respect to overall cell fluorescence. Data were combined from at least 15 cells per condition and are representative of two independent experiments. Error bars represent s.e.m. Scale bars, 10 µm. ^#^*p* < 0.05 and *p < 0.01, unpaired Student's *t*-test.

To further examine the relationship between the RAB3GAP complex and RAB18, we first investigated the localization of RAB3GAP1 in human control fibroblasts. We then compared this with the localization of RAB18 in control and RAB3GAP1(c.649-2A>G) cells. Endogenous RAB3GAP1 is known to localize to punctae overlapping with ER tubules in the cell periphery, with pronounced enrichment at a perinuclear region of Cos7 and HeLa cells [11]. It has also been reported to localize to the Golgi in human fibroblasts [16]. To determine its localization in our patient fibroblasts, we compared the pattern of staining produced by an antibody to RAB3GAP1 in control cells with that in RAB3GAP1(c.649-2A>G) cells in which the protein is absent (electronic supplementary material, figure S2*a*). Although some background staining was evident, there was clear specific signal in the perinuclear region in the control cells. RAB18 has been reported to localize to the *cis*-Golgi membrane [17,18]. To test if the ‘RAB3GAP1-specific’ signal corresponded to the *cis*-Golgi, we co-stained cells with antibodies to RAB3GAP1 and GM130 and saw clear colocalization between signals (figure 2*a*). This colocalization was not affected in either RAB18(p.Leu24Gln) or TBC1D20(p.Gln98*) cells (electronic supplementary material, figure S2*b*).

No specific signal could be identified using the available RAB18 antibodies. However, the localization of GFP-RAB18 has been shown to mirror that of its endogenous counterpart [18]. We therefore proceeded by transiently transfecting cells to express this fusion protein and imaging only cells expressing it at a relatively low level. In both control and RAB3GAP1(c.649-2A>G) cells, RAB18 showed a characteristic reticular localization pattern with enrichment at the perinuclear region. This is consistent with previous studies in which it was shown to localize to the ER membrane as well as to the *cis*-Golgi [17,18]. To confirm the *cis*-Golgi component of RAB18 fluorescence, we again co-stained cells for GM130 (figure 2*b*). There was clear colocalization between RAB18 and this marker in both control and RAB3GAP1(c.649-2A>G) cells.

The RAB3GAP complex functions as a RAB18GEF capable of ectopically targeting RAB18 to membranes. Knockdown of the complex in Cos7 and HeLa cells leads to the redistribution of RAB18 from cell membranes to the cytosol [11]. It was therefore surprising that in RAB3GAP1(c.649-2A>G) cells that lack RAB3GAP, there was no change in RAB18 localization. Rab proteins enter the cytosolic compartment when GDP-bound and complexed to GDI. Rab-GEFs promote more stable membrane association by reducing the proportion of GDP-bound Rab. However, Rabs in the cytosolic compartment are initially retargeted to ‘sample’ cellular membranes in a manner independent of Rab-GEFs [19]. We therefore reasoned that in the RAB3GAP1(c.649-2A>G) cells, efficient retargeting of RAB18 from the cytosol to the membrane could account for the lack of a ‘bulk-shift’ into the cytosol. Nevertheless, the loss of a RAB18GEF should be reflected by a more transient association of RAB18 with cellular membranes in these cells.

To investigate RAB18 dynamics in the RAB3GAP1(c.649-2A>G) cells, we employed fluorescence recovery after photobleaching (FRAP; figure 2*c*–*d*). Cells were transfected to express GFP-RAB18, and comparable fluorescent regions of interest (ROIs) within them were subjected to photobleaching with high-intensity laser light. The recovery of fluorescence in these regions was then recorded over time. Fluorescence recovery profiles following bleaching of tagged Rab proteins are complex functions reflecting fluorescence recovery from multiple sources [20]. A fast component of fluorescence recovery is the result of the simple diffusion of cytosolic fluorescent protein into the ROI following bleaching. Further components of recovery result from the lateral diffusion of membrane-associated fluorescent protein and the movement of the membranes themselves. In addition, fluorescence recovery is influenced by the rate of exchange of fluorescent protein between the cytosol and membrane, as discussed above.

Fluorescence recovery profiles from bleaching of GFP-RAB18 within ‘ER’ ROIs were not significantly different in control and RAB3GAP1(c.649-2A>G) cells (figure 2*c*). This suggests that in these cells at least, RAB3GAP does not affect RAB18 dynamics at the ER. However, bleaching of perinuclear, *cis*-Golgi, regions resulted in significantly and reproducibly different fluorescence recovery profiles between control and RAB3GAP1(c.649-2A>G) cells (figure 2*d*). As in bleaching at the ER, the extent of GFP-RAB18 fluorescence recovery recorded at the first post-bleaching timepoint (a scan begun approx. 0.5 s following completion of the bleaching scan) was comparable between the cell lines, suggesting that a similar fraction of GFP-RAB18 is cytosolic in these cells. However, fluorescence recovery in the RAB3GAP1(c.649-2A>G) cells then proceeded more rapidly and was more complete than in control cells. This suggests that in control cells, RAB3GAP serves to stabilize the association between RAB18 and the *cis*-Golgi, a conclusion supported by the enrichment of RAB3GAP1 at this location. In the RAB3GAP1(c.649-2A>G) cells, more rapid fluorescence recovery reflects a more transient, less stable, *cis*-Golgi association in the absence of RAB3GAP.

### 2.3. RAB18 dynamics at the endoplasmic reticulum are altered in TBC1D20-deficient human fibroblasts, mouse fibroblasts and HeLa cells

**Figure 3.**
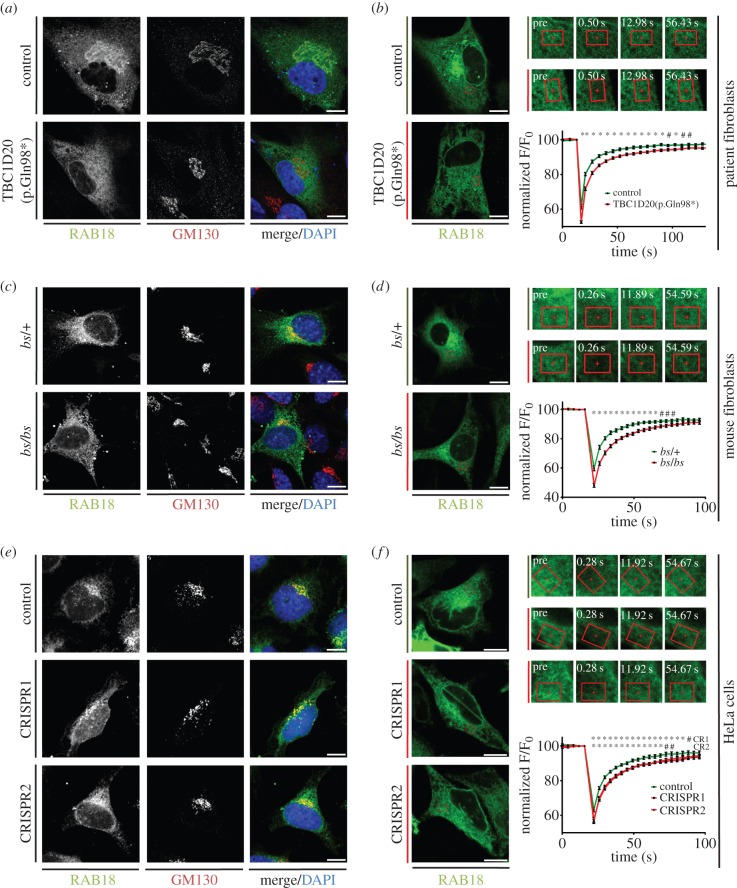
RAB18 dynamics at the ER are altered in TBC1D20(p.Gln98*) patient fibroblasts, *bs* mEFs and *TBC1D20*-deficient HeLa cells. (*a*) GFP-RAB18 colocalizes with staining for GM130 in control but not TBC1D20(p.Gln98*) fibroblasts. (*b*) GFP-RAB18 dynamics at the ER are altered in TBC1D20(p.Gln98*) fibroblasts when compared with controls. (*c*) GFP-RAB18 partly colocalizes with staining for GM130 in *bs*/+ and *bs*/*bs* mEFs. (*d*) GFP-RAB18 dynamics at the ER are altered in *bs*/*bs* compared to *bs*/+ mEFs. (*e*) GFP-RAB18 partly colocalizes with staining for GM130 in control HeLa cells and in two *TBC1D20*-deficient HeLa cell lines. (*f*) GFP-RAB18 dynamics at the ER are comparably altered in two *TBC1D20*-deficient HeLa cell lines when compared with controls. In the bleaching experiments, indicated ROIs in each cell were bleached with high-intensity laser. Fluorescence recovery in the ROI was recorded over time and normalized with respect to overall cell fluorescence. Data were combined from at least 22, 21 and 31 cells per condition in (*b*), (*d*) and (*f*), respectively, and are representative of at least three independent experiments. Error bars represent s.e.m. Scale bars, 10 µm. ^#^*p* < 0.05 and *p < 0.01, unpaired Student's *t*-test.

Having established that RAB3GAP influences RAB18 dynamics, we next investigated the relationship between TBC1D20 and RAB18. In TBC1D20(p.Gln98*) cells, the reticular ‘ER’ RAB18 staining pattern was present, but the perinuclear, *cis*-Golgi, enrichment of RAB18 fluorescence was completely absent (figure 3*a*). To confirm that RAB18 enrichment at the *cis*-Golgi was lost in TBC1D20(p.Gln98*) cells, we stained them for GM130. As shown in figure 3*a*, the clear colocalization of RAB18 and GM130 in controls was not seen in TBC1D20(p.Gln98*) cells. Together with the increase in RAB18 levels in the TBC1D20-deficient cells (figure 1*b* and electronic supplementary material, figure S1*a*), this finding strongly suggests a functional link between TBC1D20 and RAB18.

TBC1D20 is an integral ER protein with known GAP activity towards RAB18 *in vitro* [14]. Therefore, the loss of Golgi RAB18 enrichment in TBC1D20(p.Gln98*) cells suggests that TBC1D20-stimulated RAB18 GTP-hydrolysis promotes its retargeting to the Golgi. Without TBC1D20-stimulated RAB18 GTP-hydrolysis at the ER, the fraction of the protein that is GDP-bound, and therefore the fraction that is subject to GDI-mediated membrane extraction, is reduced. In turn, a smaller fraction of RAB18 in the cytosolic compartment limits its targeting to, and association with, the Golgi membrane.

The fluorescence recovery profile of RAB18 was significantly different in control and TBC1D20(p.Gln98*) cells (figure 3*b*). Fluorescence recovery at the first recorded post-bleaching timepoint was lower in the TBC1D20(p.Gln98*) cells, suggesting that in these cells a reduced fraction of GFP-RAB18 is cytosolic. Further, recovery proceeded at a slower rate and was less complete than in controls. This suggests that RAB18 is more stably ER-associated in these cells, as predicted if TBC1D20 functions as a RAB18GAP. Because TBC1D20 has previously been characterized as a GAP for Rab1 isoforms [14,21], we carried out FRAP experiments with GFP-RAB1A to test whether altered dynamics could be observed. However, we found that GFP-RAB1A fluorescence recovery was very rapid in both control and TBC1D20(p.Gln98*) fibroblasts and we were therefore unable to distinguish recovery profiles (electronic supplementary material, figure S3).

To determine whether the altered localization and dynamics of RAB18 seen in patient fibroblasts represent a consistent feature of cells lacking TBC1D20, we examined RAB18 in the *bs* mEFs (figure 3*c*). Interestingly, we found that the perinuclear enrichment of RAB18 was less pronounced in both *bs*/+ and *bs*/*bs* cells than in the human fibroblasts. Further, the colocalization between RAB18 and GM130 was less complete and did not differ between *bs*/+ and *bs*/*bs* cells. FRAP experiments, however, revealed that as in the human fibroblasts, RAB18 was less cytosolic and more stably ER-associated in the TBC1D20-deficient cells (figure 3*d*). Thus, TBC1D20 promotes dissociation of RAB18 from the ER membrane in both cell types.

It was possible that redistribution of RAB18 was not seen in the *bs*/*bs* mEFs because of some residual activity of the mutant TBC1D20 protein. To explore this possibility and also to examine RAB18 dynamics in an otherwise isogenic background, we used CRISPR technology [22] to target *TBC1D20* in HeLa cells. In order to guard against potential off-target effects, we used Cas9 nickase to induce paired single-strand breaks in the gene. Further, we used two pairs of guide RNAs to produce one cell line in which *TBC1D20* was mutated in exon 5, and one cell line in which it was mutated in exon 7. For both cell lines, cloning of the targeted exons showed that the gene was mutated and western blotting indicated that TBC1D20 protein was absent (electronic supplementary material, figure S4). As with the *bs*/*bs* mEFs, we found that loss of TBC1D20 in the HeLa cell lines did not lead to an appreciable change in RAB18 localization (figure 3*e*). Once again, however, RAB18 dynamics were altered indicating its more stable ER-association in mutant compared with control cells (figure 3*f*). The reproducibility of the FRAP approach is illustrated by the fact that fluorescence recovery profiles from the two CRISPR mutant cell lines are almost identical (figure 3*f*).

### 2.4. A reduced fraction of cellular RAB18 is cytosolic in TBC1D20-deficient human fibroblasts, mouse fibroblasts and HeLa cells

Transient knockdown of TBC1D20 in Cos7 cells has been shown to cause redistribution of RAB18 within the ER and an ‘ER sheet spreading’ phenotype [11]. This is a potential confounding factor in interpretation of our FRAP experiments, because a change in ER structure in the absence of TBC1D20 might underlie the apparent change in RAB18 dynamics. We view this as unlikely, because similarly altered ER structure in RAB3GAP1(c.649-2A>G) cells [11] had no significant effect on RAB18 dynamics (figure 2*c*). However, we nevertheless carried out additional experiments to determine more directly whether the loss of RAB3GAP or TBC1D20 affects the proportion of RAB18 that is cytosolic.

**Figure 4.**
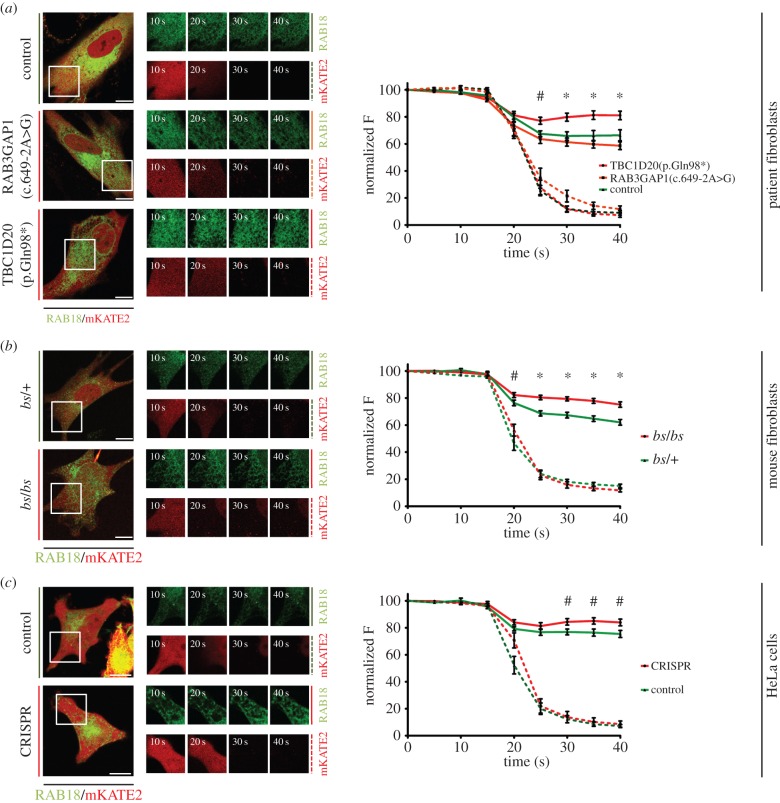
The proportion of cytosolic RAB18 in TBC1D20(p.Gln98*) patient fibroblasts, *bs* mEFs and *TBC1D20*-deficient HeLa cells is reduced compared to controls. (*a*) Loss of cytosolic GFP-RAB18 and mKATE2 fluorescence following permeabilization of human fibroblasts with 10 µM digitonin. Partial loss of GFP-RAB18 fluorescence is comparable in control and RAB3GAP1(c.649-2A>G) cells but reduced in TBC1D20(p.Gln98*) cells. mKATE2 fluorescence is reduced close to background levels in each cell line. (*b*) Partial loss of GFP-RAB18 fluorescence following digitonin permeabilization is reduced in *bs*/*bs* mEFs when compared with *bs*/+ mEFs (*c*) Partial loss of GFP-RAB18 fluorescence following digitonin permeabilization is reduced in *TBC1D20*-deficient HeLa cells when compared with controls. Data were combined from at least 10, 22 and 14 cells per condition in (*a*), (*b*) and (*c*), respectively, and are representative of at least three independent experiments. Error bars represent s.e.m. Scale bars, 10 µm. ^#^*p* < 0.05 and *p < 0.01, unpaired Student's *t*-test.

Cells were cotransfected to express GFP-RAB18 and the cytosolic red fluorescent protein mKATE2. Live cells were then permeabilized with 10 µM digitonin and the drop in fluorescence resulting from diffusion of these proteins out of cells was recorded. mKATE2 fluorescence diminished rapidly following permeabilization and was reduced to a level comparable to background fluorescence. In contrast, RAB18 fluorescence was only partially reduced, reflecting the loss of the cytosolic component, but the retention of the membrane-associated component of fluorescence (figure 4).

In the patient fibroblasts, the RAB18 loss following digitonin permeabilization was comparable in control and RAB3GAP1(c.649-2A>G) cells, whereas that in TBC1D20(p.Gln98*) cells was significantly less pronounced (figure 4*a*). RAB18 fluorescence loss was also significantly less pronounced in TBC1D20-deficient mouse fibroblasts (figure 4*b*) and HeLa cells (figure 4*c*) compared with their respective controls. These data confirm that RAB18 is less cytosolic in the absence of TBC1D20, indicating that TBC1D20 promotes dissociation of RAB18 from the ER membrane and therefore supporting the suggestion that TBC1D20 functions as a RAB18GAP.

### 2.5. A model for RAB18 regulation

**Figure 5.**
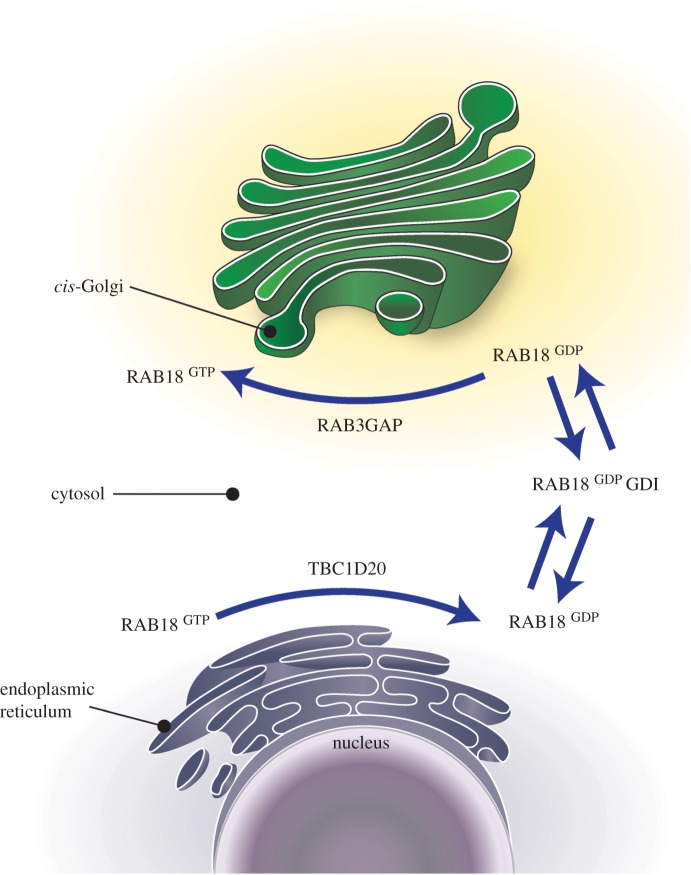
A model for RAB18 regulation. Endoplasmic reticulum (ER)-associated RAB18 hydrolyses bound GTP in a reaction potentiated by TBC1D20 activity. The resulting GDP-bound RAB18 is susceptible to GDP dissociation inhibitor (GDI)-mediated extraction into the cytosol. Cytosolic RAB18 complexed to GDI can be targeted to cellular membrane compartments including the ER and the *cis*-Golgi. GDP-bound RAB18 associated with the *cis*-Golgi can exchange bound GDP for GTP in a reaction catalysed by the RAB3GAP complex.

In this study, we examined the influence of the RAB3GAP complex and TBC1D20 on RAB18 levels, localization and dynamics. The data suggest a regulatory cycle whereby TBC1D20 RAB18GAP activity can act to promote RAB18 membrane extraction from the ER and retargeting to the *cis*-Golgi, where RAB3GAP/RAB18GEF can act to recruit and stabilize it (figure 5). RAB18 has recently been shown to interact with components of the ‘NRZ complex’, an ER tethering complex, and so this scheme is consistent with suggestions that it functions in Golgi-to-ER trafficking [23]. Interestingly, we also show that RAB18 accumulates in cells lacking RAB3GAP or TBC1D20, suggesting that normal RAB18 function, its cycles of GTP-binding and hydrolysis and/or its effector interactions are coupled to its degradation.

The clinically indistinguishable effects of loss-of-function mutations in *RAB3GAP1*, *RAB3GAP2*, *TBC1D20* or *RAB18* imply that correctly regulated GTP-binding and hydrolysis are equally essential for RAB18 function. In the broader context, this example may serve to distinguish Rab protein regulation from that of other small GTPases such as Ras. Mutations in the RasGAP *NF1*, for example, cause disease as a result of sustained Ras activation suggesting that Ras GTP-binding is sufficient for activation of downstream signalling [24]. In contrast, it appears that the downstream function of RAB18 is equally dependent on RAB3GAP/RAB18GEF and TBC1D20/RAB18GAP activity.

Primarily, our data suggest that Warburg Micro syndrome, whether caused by mutations in *RAB3GAP1*, *RAB3GAP2*, *TBC1D20* or *RAB18,* results from RAB18 dysfunction. It remains to be determined how RAB18 dysfunction contributes to disease pathology at a molecular level, but it is hoped that research in this area will continue.

## 3. Methods

### 3.1. Antibodies and reagents

Polyclonal rabbit antibodies to RAB3GAP1, used for western blotting and immunofluorescence, respectively, were obtained from Bethyl laboratories and Proteintech. A rabbit polyclonal antibody to TBC1D20 was obtained from Prestige Antibodies (Sigma). A custom rabbit polyclonal antibody to RAB18 was generated by Eurogentec to the peptide sequence N-CESENQNKGVKLSHRE-C. This antibody is specific in western blotting as it recognizes a protein of the appropriate size in samples from *wild-type* and *Rab18*+/− mice, but not in samples from *Rab18−*/− mice [12]. A rabbit polyclonal antibody to RAB1A and a goat polyclonal antibody to β-tubulin were obtained from Abcam, and monoclonal mouse anti-GM130 was obtained from BD Biosciences. Plasmids used for expression of GFP-RAB18 and GFP-RAB1A have been previously described [11,13]. The plasmid used for expression of mKATE2 was obtained from Evrogen. Cycloheximide was obtained from Abcam.

### 3.2. Quantitative PCR

For each qPCR experiment, RNAs were purified from each cell line using the Qiagen RNAeasy kit according to manufacturer's instructions. cDNAs were then produced immediately using a first strand cDNA synthesis kit (Roche). qPCR analysis was carried out on a LightCycler 480 instrument (Roche). PCR amplification was quantified through binding of specific mono colour hydrolysis probes (Roche) and analysed using LightCycler 480 software v. 1.5.0 (SP4; Roche). Primers amplifying across exon boundaries were designed using the coding sequences of *RAB3GAP1*, *TBC1D20*, *RAB18* and *Rab18* (NCBI reference sequence accessions NM_012233.2, NM_144628.3, NM_021252.4 and NM_181070.6) using the Universal ProbeLibrary Assay Design Center (Roche). Primers are listed in the electronic supplementary material, table S1.

### 3.3. Western blotting

Human fibroblasts, *bs* mEFs and HeLa cells were seeded on 6-well plates and allowed to grow for 48 h. They were then trypsinized, washed and lysed on ice in a lysis buffer containing 0.5% (v/v) Nonidet P-40 in a solution of 150 mM NaCl, 10 mM EDTA and 50 mM Tris–HCl (pH = 7.5) to which a protease inhibitor cocktail (Roche) was added. Following protein quantification, samples were combined with a reducing loading buffer and subjected to SDS–PAGE and western blotting carried out according to standard methods. Each lane on the blots shown corresponds to an individual lysate sample.

### 3.4. Cell culture and transfections

Human fibroblasts were cultured in DMEM (Gibco) supplemented with 20% fetal calf serum (FCS) and 1% penicillin/streptomycin. *bs* mEFs and HeLa cells were cultured in a similar medium containing 10% FCS. Human fibroblasts and *bs* mEFs were maintained under hypoxic conditions (3% O_2_, 5% CO_2_) at 37°C. HeLa cells were maintained under normoxic conditions (5% CO_2_) at 37°C.

Transfection of human fibroblasts and HeLa cells was carried out using Lipofectamine 2000 (Life Technologies) according to manufacturer's instructions. Briefly, up to 0.5 µg plasmid DNA was combined with 1.5 µl Lipofectamine 2000 per transfection and added to cells in a total volume of 500 µl opti-MEM serum-free media (Gibco) for 4 h. Transfection mixes were then replaced with full media, and cells were allowed to recover for 18–24 h prior to fixation or imaging. Transduction of *bs* mEFs was carried out using the Neon system (Life Technologies) according to manufacturer's instructions. Briefly, 0.5 µg plasmid DNA was combined with cells in suspension in a total of 10 µl buffer ‘R’ per electroporation and loaded into a 10 µl electroporation tip. Cells were electroporated with a single pulse at 1350 V with a width of 30 ms and allowed to adhere and recover for 18–24 h in full media prior to fixation or imaging.

### 3.5. Immunofluorescence

Cells were seeded on glass coverslips in 24-well plates, at a density of 1 × 10^5^ cells per well, then allowed to adhere overnight and transfected as appropriate. They were then fixed for 30 min with 4% (w/v) paraformaldehyde in PBS on ice. Prior to the addition of antibody, coverslips were blocked in PBS containing 10% (v/v) donkey serum (Sigma-Aldrich) and 0.1% (v/v) Triton-X-100 (Sigma-Aldrich) for 1 h at room temperature. Coverslips were probed with primary antibodies (see above) in blocking buffer, overnight at 4°C. They were then washed with PBS, and probed with Alexa Fluor 488 or Alexa Fluor 594-conjugated donkey anti-mouse or anti-rabbit secondary antibody as appropriate (Life Technologies) for 1 h at room temperature in blocking buffer. Following an additional round of washing, they were mounted and stained with DAPI prior to imaging.

### 3.6. Confocal microscopy

Imaging was carried out on a Nikon A1R confocal microscope equipped with the Nikon Perfect Focus System using a 60× oil immersion objective with a 1.4 numerical aperture. In immunofluorescence experiments, the pinhole was set to airy1. DAPI was excited using a 403.5 nm laser, and emitted light was collected at 425–475 nm. Alexa Fluor 488 or GFP was excited using a 488 nm laser, and emitted light was collected at 500–550 nm. Alexa Fluor 594 was excited using a 561.3 nm laser, and emitted light was collected at 570–620 nm.

In FRAP and digitonin-permeabilization experiments, cells were seeded onto glass-bottomed dishes (Mattek) and imaged 18–24 h post-transfection. Dishes of control and mutant cells were alternated over the course of imaging. The pinhole was set to airy2 and digital zoom parameters were kept constant for each cell type. In bleaching experiments, cells were maintained in HBSS, and bleaching was carried out using 90% laser power. In permeabilization experiments, cells were maintained in a ‘cytosol-like’ buffer containing 20 mM HEPES (pH = 7.05), 140 mM KCl, 10 mM KH_2_PO_4_, 5 mM MgCl_2_, 5.5 mM glucose and 100 mM ATP. Cells were permeabilized with 10 µM digitonin, and fluorescence loss recorded from ROIs drawn to exclude nuclei and ‘perinuclear’ GFP-RAB18.

### 3.7. Generation of cell lines

Primary mouse embryonic fibroblasts from *blind-sterile* (*bs*) mice were generated and maintained as previously described [6]. *bs*/+ and *bs*/*bs* lines were transfected with the Simian virus-40 large-T antigen (SV40) vector pBSSVD2005 (Addgene) using Lipofectamine 3000 (Life Technologies) according to manufacturer's instructions. Primers used to determine genotype at the *bs* locus and incorporation of SV40 are listed in the electronic supplementary material, table S1.

In order to specifically target *TBC1D20* in HeLa cells with Cas9 nickase, pairs of guide RNAs were selected using the online CRISPR design tool (http://crispr.mit.edu/). Oligonucleotide pairs incorporating these guide RNA sequences (see the electronic supplementary material, table S1) were then annealed and ligated into pX461 and pX462 plasmids (Addgene). Sequences were verified by direct sequencing. HeLa cells were then cotransfected with the recombinant plasmids. Each recombinant plasmid drives expression of Cas9 nickase, one guide RNA, and GFP or a puromycin-resistance gene, respectively. Twelve hours following transfection, cells were treated with 1 µg ml^−1^ puromycin for 24 h and then allowed to recover for a further 12 h. They were then trypsinized and single, cotransfected, GFP-expressing cells were sorted into 96-well plates using a FACSAria2 SORP instrument (BD). After sufficient growth, clones were analysed by PCR and western blotting.

## Supplementary Material

Supplementary Material
