# Supplementary material for: Warburg Micro syndrome is caused by RAB18 deficiency or dysregulation
Source: Open Biol. 2015 Jun 10;5(6):150047. doi: 10.1098/rsob.150047 (PMC4632505; doi:10.1098/rsob.150047)
Supplement: Supplementary Material [file rsob150047supp1.pdf]

Supplementary material

Warburg Micro syndrome is caused by RAB18 deficiency or dysregulation

Mark T. Handley [1], Sarah M. Carpanini[2], Girish R. Mali[1], Duska J. Sidjanin[3], Irene A. Aligianis[1], Ian J. Jackson[1] and David R. Fitzpatrick[1].

1. MRC Human Genetics Unit, Institute of Genetics and Molecular Medicine, University of Edinburgh, Edinburgh, Scotland, UK
2. Division of Neurobiology, The Roslin Institute and R(D)SVS, University of Edinburgh, Easter Bush, Midlothian, Scotland, UK
3. Department of Cell Biology, Neurobiology, and Anatomy, Medical College of Wisconsin, Milwaukee, USA

Address correspondence to Mark Handley: mark.handley@igmm.ed.ac.uk

Keywords: Rab18/Rab/Ras/GAP/GEF

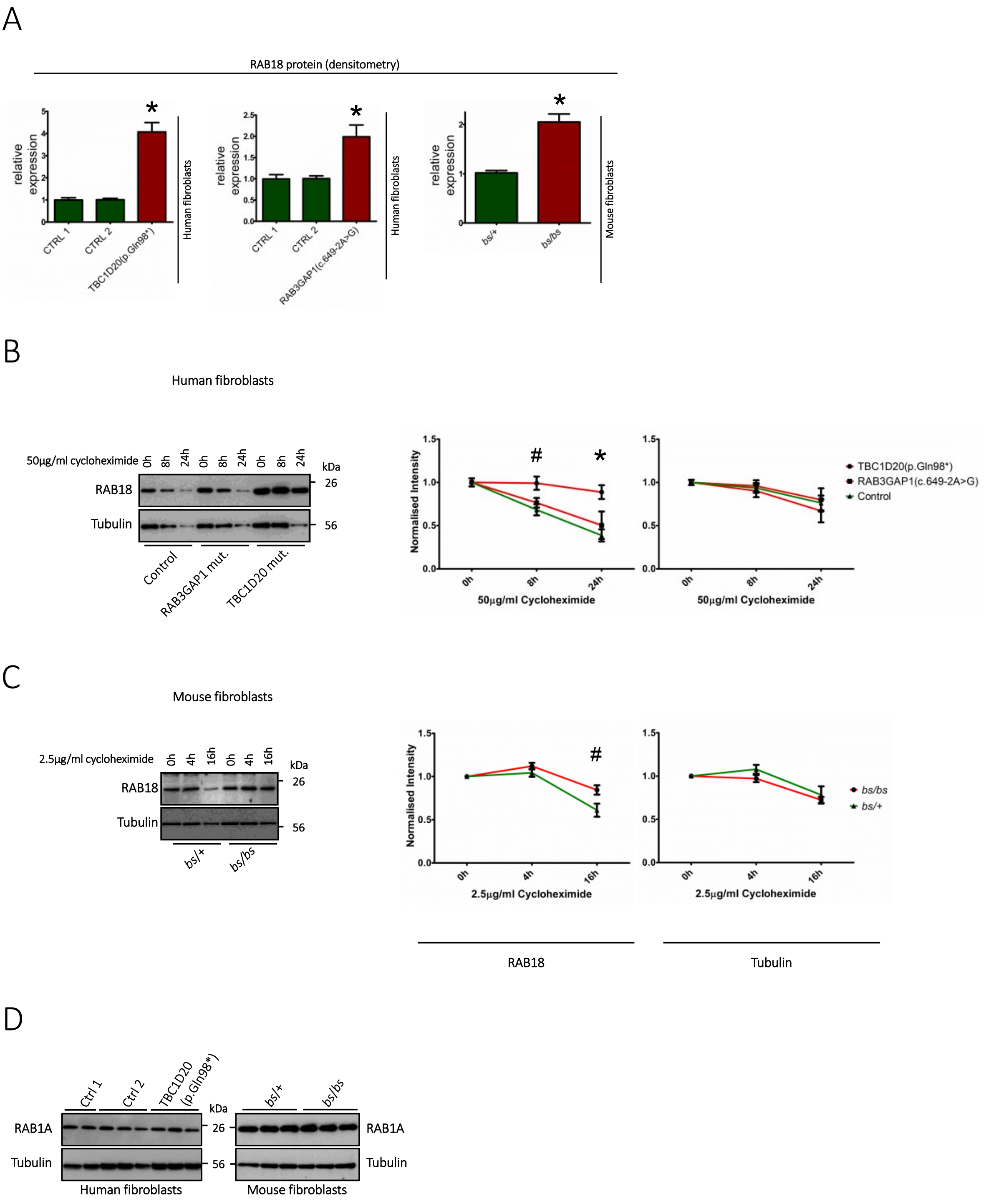

Supplementary Figure 1. RAB18 accumulation in patient fibroblasts and *blind-sterile* mEFs. (A) Levels of RAB18 signal on Western blots of control and mutant cell lysates quantified by densitometry. RAB18 signals were calculated with respect to loading controls. Data were combined from at least three independent experiments by normalising band intensity of controls to 1. (B) Cycloheximide treatment of patient fibroblasts. Control and

patient fibroblasts were treated with 50µg/ml cycloheximide for the indicated times. Levels of RAB18 and tubulin signal on Western blots of cell lysates were calculated with respect to untreated cells of the same genotype. Data were combined from at least five lysates per condition from two independent experiments. (C) Cycloheximide treatment of *bs*/+ and *bs*/*bs* mEFs. mEFs were treated with 2.5µg/ml cycloheximide for the indicated times. Levels of RAB18 and tubulin signal on Western blots of cell lysates were calculated with respect to untreated cells of the same genotype. Data were combined from at least seven lysates per condition from two independent experiments. (D) Western blotting shows comparable levels of RAB1A in lysates from control and TBC1D20(p.Gln98*) fibroblasts and from *bs*/+ and *bs*/*bs* mEFs. Blotting for Tubulin serves as a control. Each lane on the blots shown corresponds to an individual lysate sample. Band intensities were quantified using ImageJ. $^{\#}p<0.05$, $^{*}p<0.01$; unpaired Student's t test.

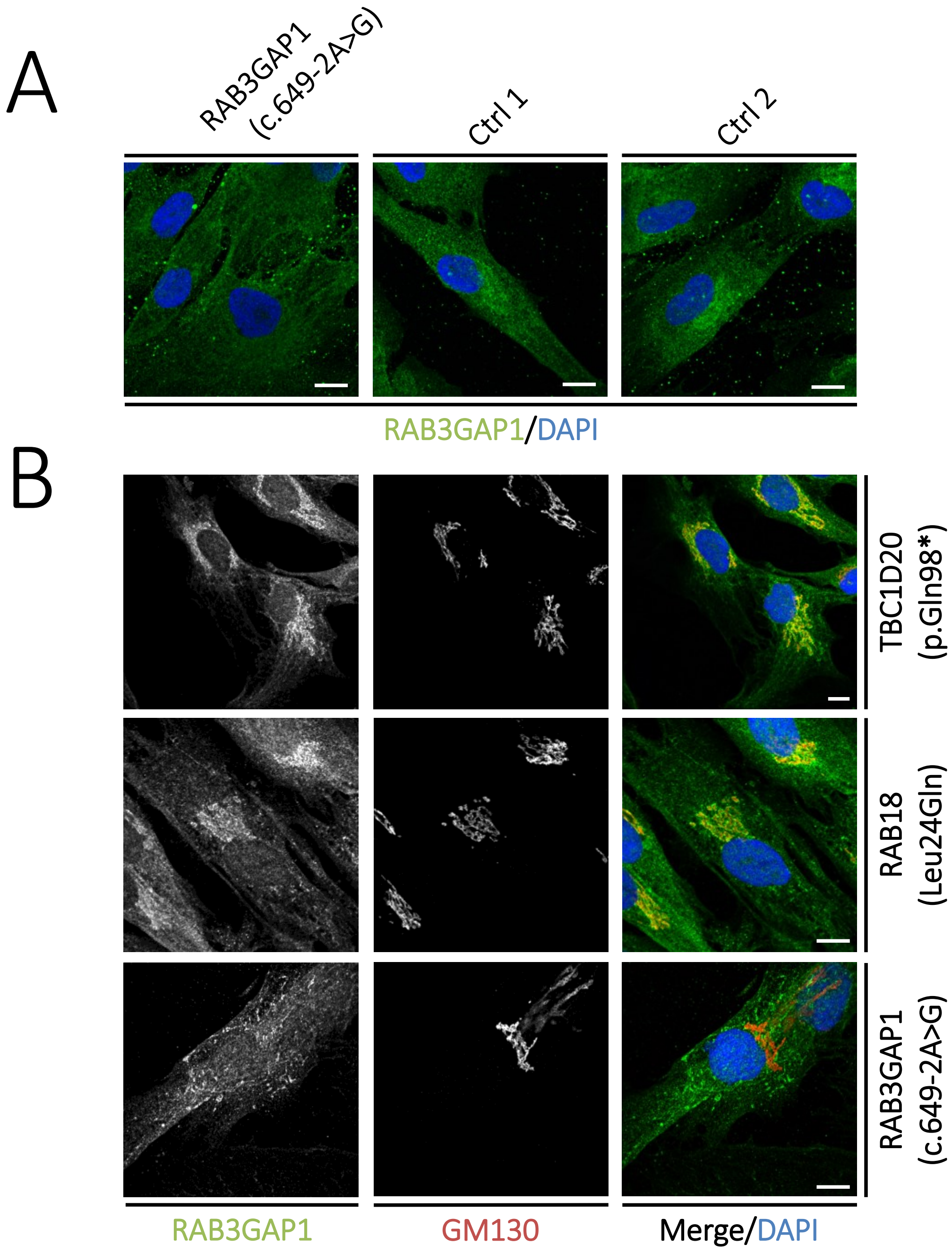

Supplementary Figure 2. RAB3GAP1 colocalises with GM130 in human fibroblasts. (A) Staining for RAB3GAP1 in RAB3GAP1-deficient fibroblasts (RAB3GAP1(c.649-2A>G) cells) produces a pattern of background staining whereas staining is enriched at the perinuclear region of control cells. (B) Staining for RAB3GAP1colocalises with staining for GM130 in TBC1D20(p.Gln98*) and RAB18(p.Leu24Gln) cells but not in RAB3GAP1(c.649-2A>G) cells. Scale bars = 10 µm.

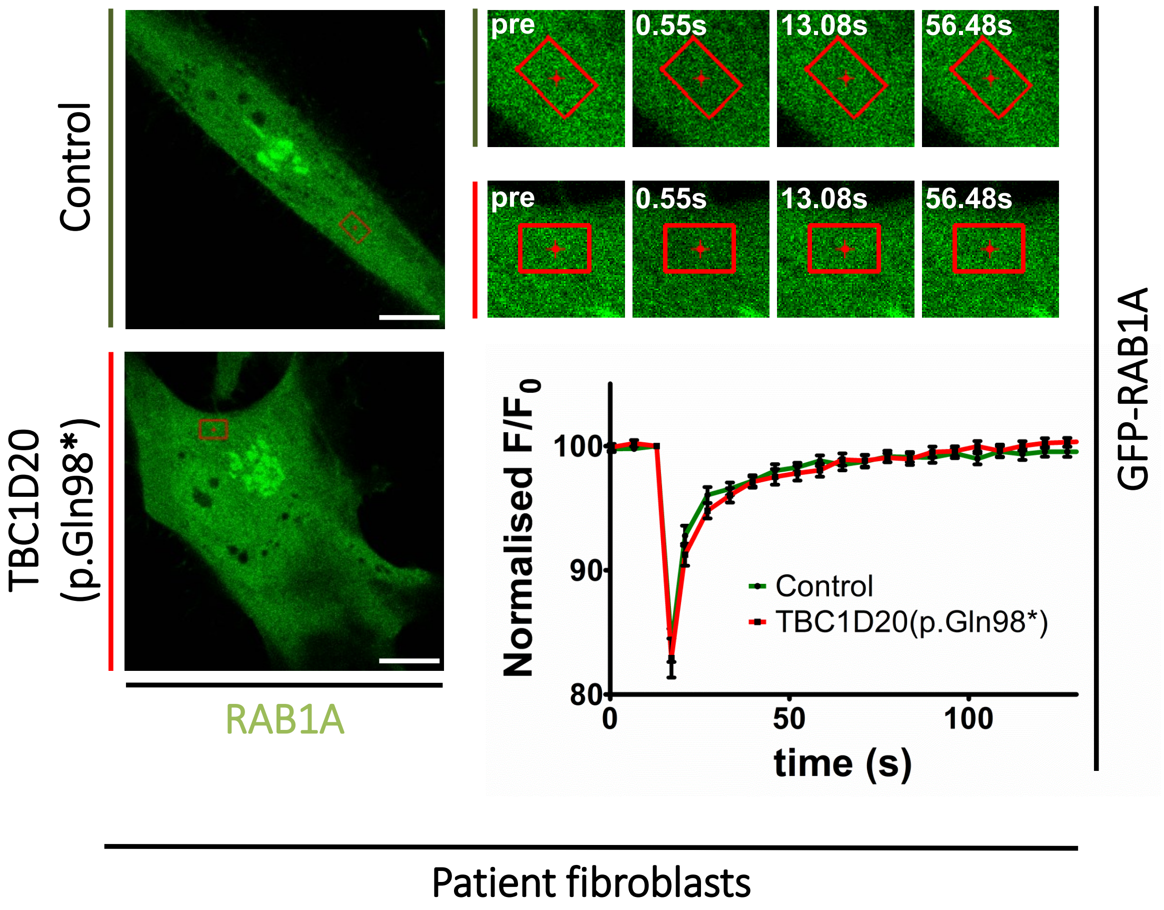

Supplementary Figure 3. RAB1A dynamics in control and TBC1D20(p.Gln98*) fibroblasts. GFP-RAB1A dynamics at the endoplasmic reticulum are comparable in control and TBC1D20(p.Gln98*) fibroblasts. Indicated regions of interest (ROIs) in each cell were bleached with high intensity laser. Fluorescence recovery in the ROI was recorded over time and normalised with respect to overall cell fluorescence. Data were combined from at least 15 cells per condition and are representative of two independent experiments. Error bars represent s.e.m. Scale bars = 10 µm.

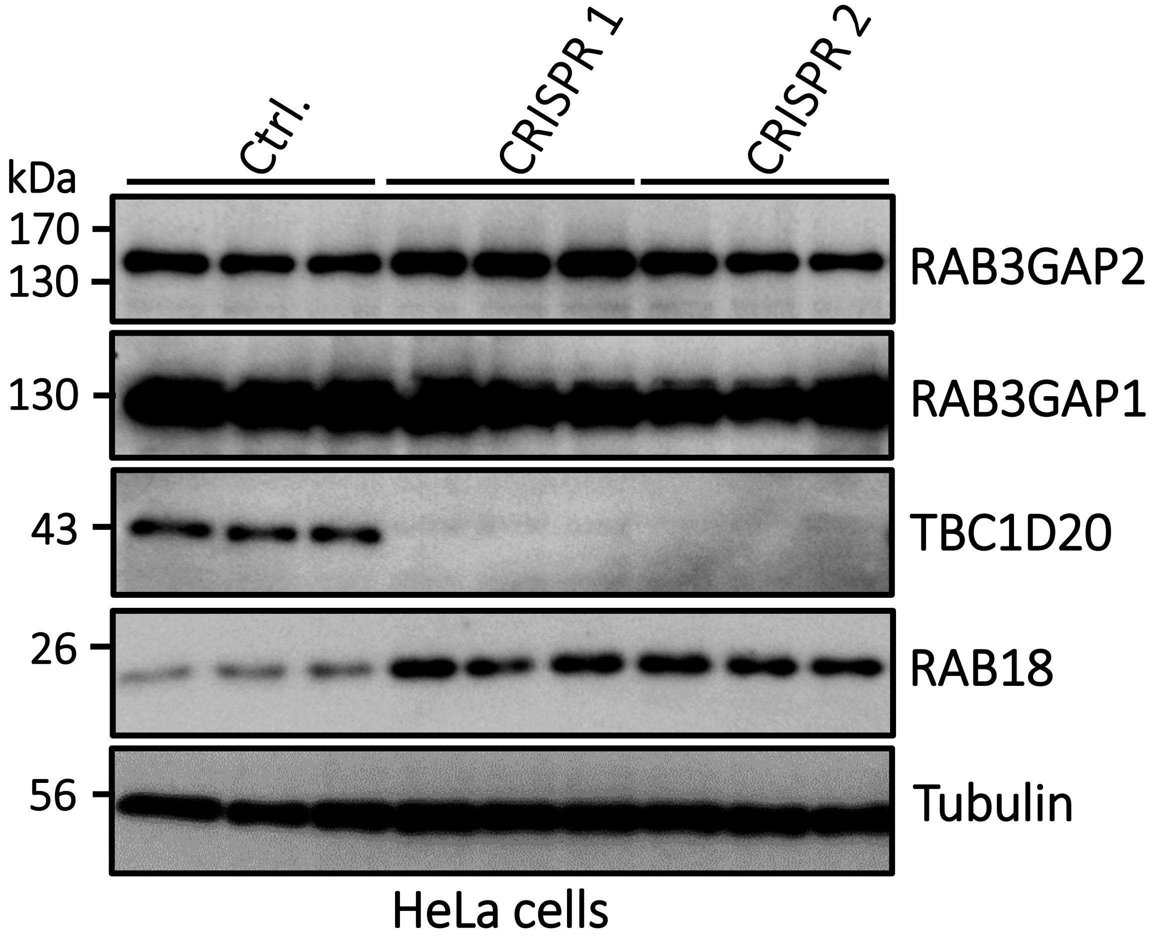

Supplementary Figure 4. Characterisation of *TBC1D20*-deficient HeLa cells. Western blotting shows that TBC1D20 protein is undetectable in CRISPR 1 and CRISPR 2 HeLa cell lines. *TBC1D20* was disrupted through transient expression of Cas9 nickase and paired guide RNAs targeting exons 5 and 7 of the gene respectively and each line was grown from a single cell. Cloning of the CRISPR 1 *TBC1D20* exon 5 locus identified c.553_591dup and c.534_565del mutations. Cloning of the CRISPR 2 *TBC1D20* exon 7 locus identified c.899_926del, c.863_893del and c.896ins50 mutations. No *wild-type* allele was cloned from either cell line.

Supplementary table 1.

qPCR primers	
Hs_TBC1D20_probe10_L	TGAGATCAGACGAAAAGTGTGG
Hs_TBC1D20_probe10_R	CATCTGCCGTAGGTTCTTCC
Hs_RAB3GAP1_probe2_L	GGAGTCACCGCTAAATAATGATG
Hs_RAB3GAP1_probe2_R	TCAGAAACAGCATCAGGGAAT
Hs_RAB18_probe33_L	CTGGTCAAGAGAGGTTTAGAACATT
Hs_RAB18_probe33_R	CAAATGTATCTCTTCTTGTGACATCAT
Hs_TBP_Probe3_L	CGGCTGTTTAACTTCGCTTC
Hs_TBP_Probe3_R	CACACGCCAAGAAACAGTGA
Hs_beta_actin_Probe64_L	CCAACCGCGAGAAGATGA
Hs_beta_actin_Probe64_R	CCAGAGGCGTACAGGGATAG
Ms_RAB18_probe33_L	GCTGGTCAAGAGAGGTTCAGA
Ms_RAB18_probe33_R	GGTGTCTCTTCTTGTGACATCATAG
Ms_GAPDH_probe52_L	GGGTTCCTATAAATACGGACTGC
Ms_GAPDH_probe52_R	CCATTTTGTCTACGGGACGA
CRISPR oligos	
CRISPR_TBC1D20_exon5_G1_TOP	CACCGGTGCTTGGTGTTGTCCATTGT
CRISPR_TBC1D20_exon5_G1_BOTTOM	AAACACAATGGACAACACCAAGCACC
CRISPR_TBC1D20_exon5_G2_TOP	CACCGGTCTGATGCCCATCATTGACC
CRISPR_TBC1D20_exon5_G2_BOTTOM	AAACGGTCAATGATGGGCATCAGACC
CRISPR_TBC1D20_exon7_G1_TOP	CACCGGCTGCTGATCAGTGTCTCATA
CRISPR_TBC1D20_exon7_G1_BOTTOM	AAACTATGAGACACTGATCAGCAGCC
CRISPR_TBC1D20_exon7_G2_TOP	CACCGGCCCATCCGAACTTGCTCGGG
CRISPR_TBC1D20_exon7_G2_BOTTOM	AAACCCCGAGCAAGTTCGGATGGGCC
sequencing/cloning primers	
Hs_TBC1D20_exon5_F	GTAGCGCGACGGCCAGTACAATGAGGAAACTGCGCTC
Hs_TBC1D20_exon5_R	CACCAACTCACTGGCCTGTTTTCTG
Hs_TBC1D20_exon7_F	CACCCCAACCCTATCCCACCAG
Hs_TBC1D20_exon7_R	CAGGGCGCAGCGATGACAACATTTGGGCTGAGTCCTG
bs_F	CAGCTGGCTTATCACCTGGTTTG
bs_R	TGCAAAGTAAATGGGCATAAGT
SV40_F	TAATACGACTCACTATAGGG
SV40_R	GAATGTAAAGGGCACTGGAG
